# Progress in Psychiatry

**Published:** 1900-04-21

**Authors:** 


### Progress in Psychiatry.

(*Continued from page 327, Vol. XXVII.*)

**Insanity and Race Decay.**—The problems relating to insanity as considered in previous chapters have practical interest. The great biological advances of the century, which have borne such splendid fruit in all branches of knowledge, have done singularly little in enlightening us on the various conditions and causes of race decay and family degeneration. Yet these causes demand attention and investigation, for they sap at the very foundations of human society.

Insanity, like vice and crime, is in no sense an accident in human civilisation. It has a positive pathological aspect, a dangerous vitality which enables it under favourable conditions to multiply and grow beyond healthy limits, to migrate and get disseminated through the community until remedial measures are imperatively called for. The causes of insanity include everything that is inimical to a healthy and well-balanced life—physical, moral, and social. That such causes should act more potently on some members of the community than on others is a necessity born of the very laws of human life, an eventuality which is bound to occur in nearly every form of human society.

Indeed, were all cases of insanity blotted out from the existing generation, fresh contingents would arise in the next beyond a doubt.

Nevertheless there exists a group of ætiological factors, briefly referred to already (page 211), which medical and social science is now beginning to recognise as of great importance and partially preventable. These factors are early surroundings of chronic poverty and want, deficient food and clothing, anæmia and overcrowding, early associations of vice and crime, and inability to rise above the margin of starvation, all of which constitute a complex but most potent factor in the production of cerebral and bodily degeneracies of all sorts. Mr. Charles Booth, the statistician, has collected facts and figures which tend to show that there were 3,000,000 of people in England in 1890 (out of a population of 26,000,000), who could not live for a week on their own resources. There were 100,000 waifs and strays sleeping under hedges, on bridges, in carts, and in open spaces, with no resources at all. These comprise individuals at large, and do not include the vast pauper degenerate populations of our public asylums and workhouses. Mixed up with this floating mass of degeneracy, and indeed so intimately that it is difficult to differentiate between the two, there exists a large body of individuals, of which the males constitute a vagrant alcoholic and criminal portion, and the females an immoral and prostitute class. These dwellers on the border of the social swamp have little or no idea of hygiene, bodily or mental; while they are gifted with a capacity for free and unrestrained propagation, and this whether afflicted with the taint of actual insanity or not. The children born of such parents are stunted or ill-formed in body, feebly and erratically organised in brain, and hence capable of little intellectual and less moral development; ill-balanced and unstable weaklings prone to succumb readily to the promptings of vice and of crime, and peculiarly liable to suffer from cerebral and spinal diseases. It is from these factories of degenerate humanity that there issue forth the largest numbers of our feeble-minded, crippled, or idiotic children, our epileptic idiots and imbeciles, our congenital moral delinquents and petty thieves, our neurasthenic criminals and prostitutes. It is necessary to observe, however, that the laws of heredity when operative in such cases bring about a twofold result. In the first place, crossings with healthy blood may take place, and this may in a measure serve to arrest the progress of degeneration. In the second place—and this is more striking—crossing with tainted blood, with individuals of the same type and temperament, often takes place, with the result that a few generations of offspring appear, but with gradually diminishing vitality, so that in the course of the fourth or fifth or sixth generation the stock ends in sterile idiocy. A perusal of the two publications issued by the Charity Organisation Society ([19] and [20]), will clearly show forth some of the more important aspects of this fact. The well-known facts concerning endemic goitre, demi-cretinism, and endemic cretinism illustrate the operations of the law of degeneration in certain restricted localities of the globe, *e.g.*, Switzerland, Savoy, and the Tyrol. The race of cretins begins with healthy ancestors who, migrating into and settling in the cretin-producing valleys, beget children who become afflicted with goitre and grow weak-minded. These goitrous, weak-minded individuals are known as demi-cretins. They intermarry, and their children grow usually into fully developed cretins, imbeciles and idiots with stunted body and mind and a characteristic physiognomy and incapable of procreation as a rule.[21] Thus it happens that starting with healthy ancestors, it is possible to produce a race of cretinoid idiots and imbeciles in the third or fourth generation, with whom the family becomes extinct. Yet the race of cretins is kept up, for fresh arrivals settle in these cretin-producing localities, and the course of pathological history sketched above is repeated.

[19] The Epileptic and Crippled: London, 1893. [20] The Feeble Minded: London, 1893. [21] Art. Cretinism in Tuke's Dictionary of Psycholog. Medicine, 1892; and Morel loc. cit.

